# Knowledge Mapping of the Phytoremediation of Cadmium-Contaminated Soil: A Bibliometric Analysis from 1994 to 2021

**DOI:** 10.3390/ijerph19126987

**Published:** 2022-06-07

**Authors:** Xiaofeng Zhao, Mei Lei, Runyao Gu

**Affiliations:** 1 Institute of Geographic Sciences and Natural Resources Research, Chinese Academy of Sciences, Beijing 100101, China; zhaoxf.15b@igsnrr.ac.cn; 2 University of Chinese Academy of Sciences, Beijing 100049, China; 3 College of Eco-Environmental Engineering, Guizhou Minzu University, Guiyang 550025, China; gu.ry@gzmu.edu.cn

**Keywords:** phytoremediation, cadmium, hyperaccumulator, bibliometric analysis, Web of Science

## Abstract

Cadmium pollution of soil threatens the environmental quality and human health. Phytoremediation of cadmium-contaminated soil has attracted global attention in recent decades. This study aimed to conduct a comprehensive and systematic review of the literature on phytoremediation of cadmium-contaminated soil based on bibliometric analysis. A total of 5494 articles published between 1994 and 2021 were retrieved from the Web of Science Core Collection. Our knowledge mapping presented the authors, journals, countries, institutions, and other basic information to understand the development status of phytoremediation of cadmium-contaminated soil. Based on a keyword cluster analysis, the identified major research domains were “biochar”, “*Thlaspi caerulescens*”, “endophytic bacteria”, “oxidative stress”, “EDTA”, and “bioconcentration factor”. Overall, this study provided a detailed summary of research trends and hotspots. Based on the keyword co-occurrence and burst analysis, the core concepts and basic theories of this field were completed in 2011. However, the pace of theoretical development has been relatively slow. Finally, future research trends/frontiers were proposed, such as biochar addition, rhizosphere bacterial community manipulation, cadmium subcellular distribution, and health risk assessment.

## 1. Introduction

Soil quality directly affects food quality and human health. However, the rapid development of human society and activities such as mining, smelting, irrigation, and industry have caused significant heavy metal (HM) pollution to soil [1,2,3]. Cadmium is a non-essential element that is toxic to plants, even at a low concentration [4,5]. In humans, exposure to cadmium may increase the risk of kidney disease, skeletal damage, and even cancer [6]. According to the National Commission of Soil Pollution Survey [7], the over-standard rate of cadmium is 7.0% in China. In the European Union, 5.5% of the samples from the European Union’s first harmonized topsoil sampling had cadmium concentrations above the threshold value [8].

Phytoremediation, a cost-effective and environmentally-friendly technology, has been proven by international researchers to be an effective way to remove metals from contaminated soil [9,10]. Researchers are actively seeking out plants with the ability to hyperaccumulate cadmium in their shoots [11,12,13]. To date, extensive research has been conducted in multiple disciplines (e.g., cytology, molecular biology, and genetics) to explore the hyperaccumulation mechanisms in plants [14,15,16]. Meanwhile, various hyperaccumulator plants have been applied to remediate metal-contaminated soil, and the methods that enhance the repair effects have been explored [17,18,19].

Currently, extensive cutting-edge research has been conducted and great progress has been achieved in recent decades. Considering the extensive research efforts devoted to this research domain, there is a need to undertake a systematic analysis that could provide an overview of the current situation and potential future trends. Many review studies have been conducted to disclose the present development situations (e.g., the application and fundamentals of phytoremediation and combining different technologies to improve efficiency) [20,21,22]. However, these research review papers are mainly based on subjective evaluations that included a small number of articles. Thus, a holistic and detailed bibliometric analysis is still lacking; there is a need for a systematic and comprehensive evaluation of the current research to identify research trends and future research areas. Due to the limitations of previous literature reviews, comprehensive research frontier and development trends of phytoremediation of cadmium-contaminated soil have not yet been established, inhibiting development and innovation in this field.

The rapid development of scientometrics and informatics technology has provided more reliable methods for research on big data visualization. CiteSpace is a Java-based scientific visualization tool for citation network analysis, first launched in 2004 by Professor Chaomei Chen of Drexel University [23]. Topic areas, hotspots, and research trends can be clearly presented using co-occurrence analysis and co-cited analysis functions. A co-occurrence analysis of country, institution, and author could reveal the main contributing sources in this field. CiteSpace is an excellent bibliometric analysis software; therefore, it was selected to analyze the research trends, active institutes and authors, hotspots, and their co-occurrence in literature on phytoremediation of cadmium-contaminated soil. The results of the analysis aim to provide a more comprehensive understanding and inspiration for this field.

## 2. Materials and Methods

### 2.1. Data Collection

The Web of Science (WOS) is characterized as the most accurate literature-indexing tool for technical and scientific knowledge analysis, and it can provide insights into the frontiers of the most critical research areas. In this study, the data used in the bibliometric analysis were collected from the WOS, including the Science Citation Index-Expanded (SCI-EXPANDED), Conference Proceedings Citation Index-Science (CPCI-S), Social Sciences Citation Index (SSCI), Conference Proceedings Citation Index-Social Sciences and Humanities (CPCI-SSH), Book Citation Index-Science (BKCI-S), Art and Humanities Citation Index (A & HCI), and Index Chemicus (IC).

Phytoremediation of cadmium-contaminated soil was chosen as the research topic. The following were the search terms used in the study: TS = ((Cd OR cadmium) AND (hyperaccumulat* OR hyper-accumulator OR phytoremediat* OR phytoextract* OR phytostabilizat* OR phytodegradat* OR phytostimulat* OR phytovolatilizat* OR rhizofiltrat* OR phytodesalinat* OR phytotransformat* OR rhizodegradat* OR rhizoattenuat* OR “plant repair” OR “plant restore”) AND (soil*)). Document types were limited to articles and reviews articles. A total of 5494 publications from 1994 to 2021 were collected, which includes 5262 articles and 232 review articles.

### 2.2. Analysis Methods

CiteSpace (5.8.R3) (Drexel University, Philadelphia, PA, USA) was used for the bibliometric analysis in this study. The visualization knowledge network created by CiteSpace consists of nodes and links. The nodes in each map represent a category of elements (e.g., cited references, institutions, authors, and countries). The links between nodes represent the relationships between collaboration/cooccurrence or co-citations; the thicker the line, the closer the connection is. Furthermore, the colors of nodes and lines represent different years. Nodes with high betweenness centrality are pivotal points in CiteSpace [24]. The betweenness centrality is defined in Equation (1).

(1) $$\mathrm{Centrality}\left({\mathrm{node}}_{i}\right)=\sum_{i\neq j\neq k}\frac{\sigma_{jk}\left(i\right)}{\sigma_{jk}},$$

In Equation (1), $\sigma_{jk}$ represents the total number of shortest paths between node j and node k, and $\sigma_{jk}\left(i\right)$ is the number of paths that pass through ${\mathrm{node}}_{i}$. The purple ring represents centrality. Nodes with high centrality generally represent turning or pivotal points in a field.

Clustering algorithms are widely used in text data mining. The clustering principle is that objects in the same cluster have good similarities, whereas objects in different clusters are quite different [25]. CiteSpace provides an automatic clustering function based on the spectral clustering algorithm. The log-likelihood rate (LLR) was chosen for the clustering algorithm in this study; the larger the LLR is, the more representative the words are to this cluster [26]. The silhouette value is an indicator that can measure network homogeneity and is generally used to evaluate the clustering effect [27]. The silhouette value varies between 0 and 1, and the closer it is to 1, the higher is the network homogeneity [28].

The parameters of CiteSpace were set as follows: time slicing (1994–2021), years per slice (1), term source (all selections), node type (choose one each time), strength (cosine), scope (within slices), selection criteria (scale factor *k* = 25), and pruning (pruning sliced network). After defining the parameters, iterations were run in CiteSpace to obtain the data and corresponding networks. Related data and mapping networks were examined. Finally, the results of the visualization analyses were presented and discussed in the following section.

A variation trend graph of the number of annual publications was drawn using Origin 2021b. ArcGIS 10.7 (ESRI, Redlands, CA, USA) was used to map the global geographic distribution of the selected publications.

## 3. Results and Discussion

### 3.1. Overview of Bibliometric Characteristics

#### 3.1.1. Publication Output

**Figure 1:**
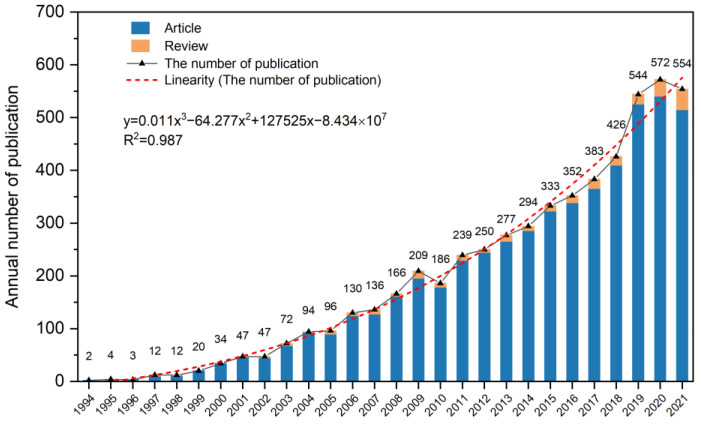
Annual publication number on phytoremediation of cadmium-contaminated soil between 1994 and 2021.

The ascending number of annual publications indicated major research progress in this field, as shown in Figure 1. The number of publications conformed to the cubic curve regression model (*y* = 0.008*x*^3^ − 48.459*x*^2^ + 95855*x* − 6.320 × 10^7^, *R*^2^ = 0.988). Rapid growth was observed over the research period. The annual number of publications increased from 2 in 1994 to 554 in 2021, and a total of 5494 articles were published during this period. Prior to 2003, the number of publications in this research field was relatively small (less than 50 per year). However, from 2006, the annual number of publications showed an upward trend and exceeded 100 publications. In 2017, the annual publications reached 383, indicating that one relevant article was published every day on average. Moreover, nearly half (45.12%) of the articles were published between 2017 and 2021. The variation trend of the annual number of publications indicated that this field had received increasing attention in the past three decades, especially in recent years.

#### 3.1.2. Categories

**Figure 2:**
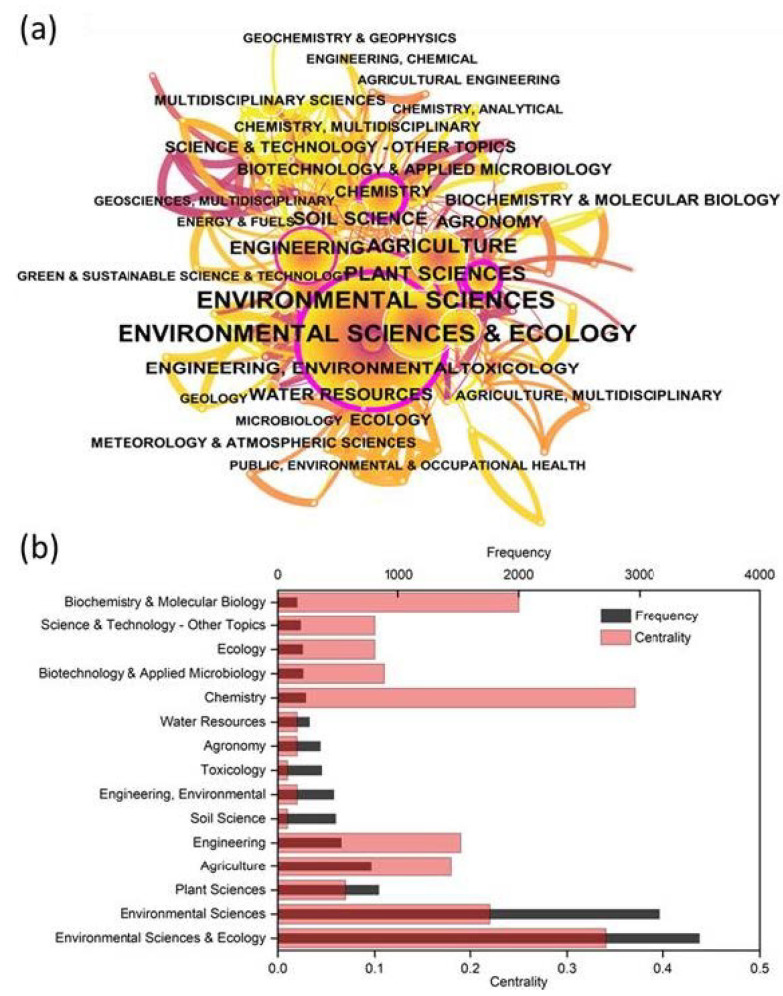
Schematic diagram of thematic categories between 1994–2021 (**a**) and topical subjects/categories with high frequency and centrality (**b**).

The co-occurrence analysis of subject categories can reveal their inter-disciplinary links and intrinsic connections [29,30]. Figure 2 shows the co-occurrence network of a subject category (Figure 2a) and the top 15 categories in terms of frequency and centrality (Figure 2b). In Figure 2a, each node represents a different subject category; the purple ring indicates the high centrality values of the corresponding categories, and the lines between the nodes demonstrate the co-occurrence of these subject categories. As shown in Figure 2a, “environmental sciences and ecology” (citation counts 3503), “environmental sciences” (3173), “plant sciences” (835), “agriculture” (771), and “engineering” (525) were the five most popular categories of research. In Figure 2b, the centrality of “chemistry” (0.37) was the largest, followed by “environmental sciences and ecology” (0.34), indicating that these two categories had a greater influence on the phytoremediation of cadmium-contaminated soil. The frequency of “environmental sciences and ecology” (3503) is larger than that of “chemistry” (232), but in terms of centrality, “environmental sciences and ecology” (0.34) is lower than “chemistry” (0.37). This indicated that the category with the highest visibility will not always be accompanied by the greatest influence [31].

#### 3.1.3. Journal Source

The 5494 publications were disseminated across 654 journals. Table 1 lists the top ten journals that published the most articles in this field. Among all the journals, the *International Journal of Phytoremediation* was the most productive journal (474 publications, 8.63%), followed by *Environmental Science and Pollution Research* (429 publications, 7.81%), and *Chemosphere* (325 publications, 5.92%). The following journals also accepted articles in this field, but each contribution was less than 5%: *Ecotoxicology and Environmental Safety* (217 publications, 3.95%), *Environmental Pollution* (189 publications, 3.44%), *Journal of Hazardous Materials* (163 publications, 2.97%), *Science of the Total Environment* (158 publications, 2.88%), *Plant and Soil* (158 publications, 2.88%), *Water Air and Soil Pollution* (117 publications, 2.13%), and *Journal of Environmental Management* (79 publications, 1.43%). Among the top ten journals, the *International Journal of Phytoremediation* mainly focused on phytoremediation; *Environmental Science and Pollution Research*, *Chemosphere*, *Ecotoxicology and Environmental Safety*, *Environmental Pollution*, *Journal of Hazardous Materials*, *Science of the Total Environment*, and *Journal of Environmental Management* were related more to environmental science (i.e., environmental pollution, environmental chemistry, and environmental safety); and *Plant and Soil* and *Water Air and Soil Pollution* were related to soil. Publications from the top ten journals together accounted for 42.03% of the total publications in this field.

### 3.2. Co-Authorship Analysis

#### 3.2.1. Author Co-Authorship Analysis

**Figure 3:**
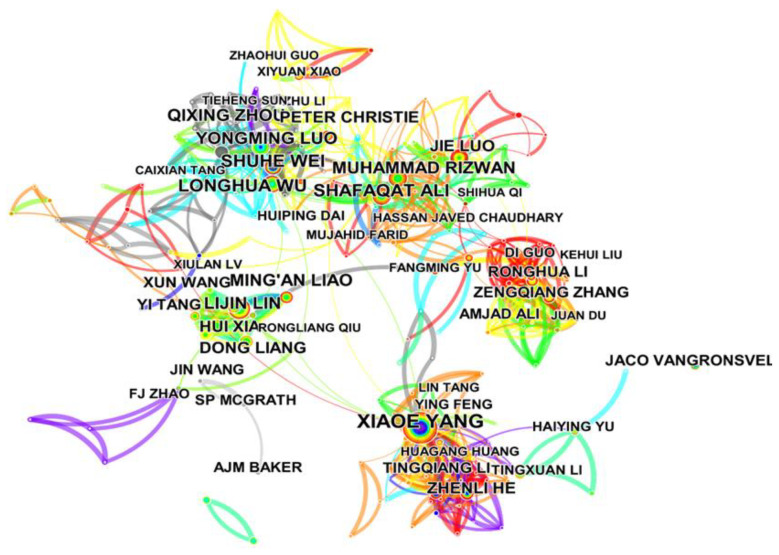
Author cooperation network.

The author cooperation analysis revealed the contribution of an author and the cooperative relationship between different authors. The author cooperation network is presented in Figure 3, where each node represents an author, and the node size are proportional to the publication number of the author. The links between the nodes suggested a cooperative relationship between these authors. The top ten authors for the number of publications are listed in Table 1. The most active author in this field was Xiaoe Yang (72 publications), followed by Shuhe Wei (42 publications), Longhua Wu (41 publications), and Shafaqa Ali (41 publications). These authors have made significant contributions to this field. Yang et al. revealed that *Sedum alfredii*, could hyperaccumulate cadmium and lead; in addition, they conducted extensive studies on the enrichment mechanism and successfully applied it in phytoremediation [13,32,33]. Shuhe Wei conducted several studies in screening new cadmium hyperaccumulators and actively applied them in phytoremediation [9,34,35]. Longhua Wu found that *Sedum plumbizincicola* had a strong ability to absorb cadmium from soil, and made serious progress in soil remediation and intercropping [36,37,38]. Shafaqa Ali conducted significant research on cadmium phytoextraction enhancement, cadmium phytoavailability reduction, and translocation of metals from soil to wheat [39,40,41]. There was a weak cluster connection between the research (Figure 3), which means that the authors tended to cooperate in relatively small-scale teams. Hence, more cooperation across international teams is required in future studies.

#### 3.2.2. Institution Co-Authorship Analysis

**Figure 4:**
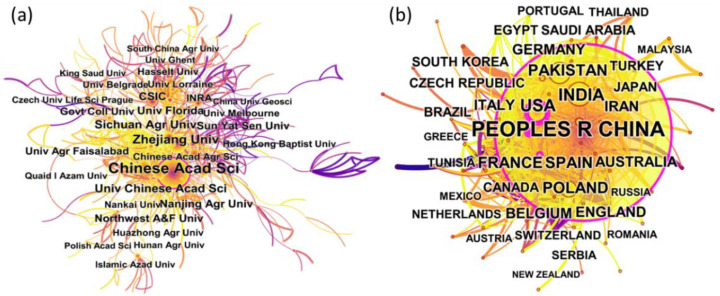
The cooperation network of institutions (**a**) and the cooperation network of countries (**b**).

The cooperation network can reveal the academic influence of the institution and country to a certain extent. Most contributing institutions are from China, the USA, Pakistan, France, Spain and Belgium. There were 8 Chinese institutions in the top 15 institutions in terms of the number of articles published. France and Pakistan had two institutions each in the top institutions. The remaining three were from the USA, Spain, and Belgium. Among these institutions, the Chinese Academy of Sciences (439 articles) and Zhejiang University (163 articles) made a significant contribution to cadmium hyperaccumulator. The University of Chinese Academy of Sciences (108), Sichuan Agricultural University (107 articles), and University of Florida (88) also conducted research in this field. From the cooperation network of institutions (Figure 4a), we observed relatively close cooperation among several institutions. For example, the Chinese Academy of Sciences, Zhejiang University, and the University of Florida had close cooperation with others, while other institutions also cooperated closely.

#### 3.2.3. Country Co-Authorship Analysis

A cooperation network of countries showed that most contributing institutions came primarily from China, and these results were similar to those of the co-authorship analysis. As shown in Figure 4b and Table 1, China (1950 articles) was the country with the highest contribution, followed by the USA (474), India (415), Pakistan (319), and France (219).

Scholars in China (1950 articles) have conducted far more research on phytoremediation of cadmium-contaminated soil than those in other countries. Several factors can be attributed to this trend. First, China is rich in plant resources, especially in broad-leaved evergreen forests in southern China, which is less affected by Quaternary glaciers; therefore, various ancient plants are retained, including 14,600 species of seed plants [42]. Second, these areas are rich in HM mineral resources; thus, the soil around some mining areas or metallogenic belts contain relatively high levels of HMs [1]. The abundant plant resources and high HM concentrations in soils have provided the basis for the evolution of hyperaccumulators. Lastly, the Chinese government has been actively improving environmental quality in recent years and has invested considerable financial and human resources in the remediation of polluted soil [43,44,45]. Therefore, phytoremediation, as a green and effective technology for the treatment of polluted soil, has received significant attention and application in China.

**Figure 5:**
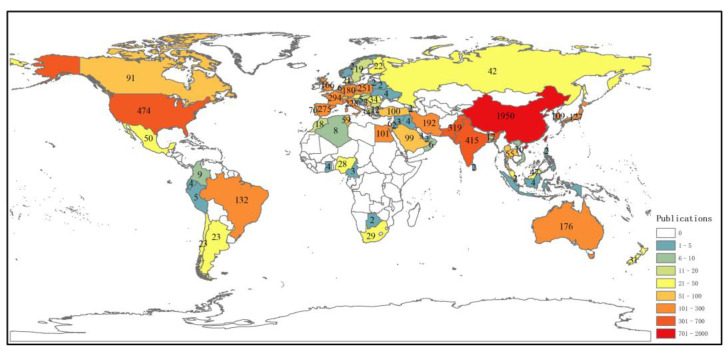
Geographic distribution of countries studying phytoremediation of cadmium-contaminated soil.

In terms of centrality (Figure 4b and Table 1), the USA (0.23), Italy (0.21), and Japan (0.15) were the three most influential countries, followed by Spain (0.14) and China (0.12). The USA was the most influential country. This may be explained by the fact that scholars in the USA had performed several pioneering research in this field in the early periods. They defined several terminologies and outlined the main considerations for hyperaccumulation [21,46,47]. These pioneering works have played a leading role and pushed forward the development of this field. From a continental aspect, productive countries are mainly located in Asia, North America, and Europe (Figure 5).

### 3.3. Co-Citation Analysis

#### 3.3.1. Journal Co-Citation Analysis

A network of journal co-citations could provide us with more innovative information about these journals. The journal co-citation network is presented in Table 1 and Figure S1a; the order from high to low frequency was *Chemosphere* (frequency = 3801), *Environmental Pollution* (frequency = 3644), and *Plant and Soil* (frequency = 3323), all of which had more than 3000 co-citation frequencies. The next were the *Science of the Total Environment* (frequency = 2745) and *Journal of Hazardous Materials* (frequency = 2592). These journals are amongst the top journals in the field. Although some journals (e.g., *Science of the Total Environment* and *Environmental Pollution*) published only a few relevant articles, they still have a profound influence in this field.

#### 3.3.2. Author Co-Citation Analysis

The author co-citation network could reflect the influence of authors in the research, and it would provide us with a complete view of the field. As shown in Table 1 and Figure S1b, the author with the highest citation frequency was Baker A.J.M. (1648 citations, Australia). Baker A.J.M., a pioneer in the field of phytoremediation, along with his colleagues, conducted numerous novel research in phytoremediation, agromining, and other relevant areas [21,48,49]. This author was followed by Salt D.E. (1009 citations), McGrath S.P. (897 citations), Kabata-Pendias A. (745 citations), and Zhao F.J. (600 citations), all of which published influential works and promoted the development of this field [50,51,52,53]. However, the authors’ citation numbers were not always consistent with their publication frequency (Table 1). This indicated that some authors made significant contributions, despite not having published many articles.

#### 3.3.3. The Top 10 Most-Cited Research Articles

The top 10 most-cited research articles were listed in Table 2. These articles were all significant and instructive for phytoremediation of cadmium-contaminated soil. They were nearly all published in authoritative journals of this field (e.g., *Environmental Science & Technology*, *Plant and Soil*, and *New Phytologist*). Some works had laid a foundation for the following research works in the field of phytoremediation. For example, Kumar et al. [47] elaborated the concept and basic principle of phytoremediation in detail, which had attracted wide attention and boosted the development of relevant works. van der Ent et al. [21] outlined the main considerations for establishing metal or metalloid hyperaccumulation status of plants, (re)defined some of the terminology and noted potential pitfalls, which contributed to the further corrections and improvements of this area.

At the same time, enhancing phytoremediation efficiencies had also attracted wide attention. Huang et al. [54] introduced the role of synthetic chelates in lead phytoextraction. This finding had aroused great repercussions, and chelates addition had become a hotspot. Additionally, some studies had expanded the application of hyperaccumulators. Dushenkov et al. [55] reported that accumulating-plants can remove heavy metals from aqueous streams; Stoltz et al. [56] reported that accumulating-plants can also reduce element leakage from submerged mine tailings. Moreover, Baker et al. [11], Yang et al. [13], Brown et al. [57], Dahmani-Muller et al. [58], and Ebbs et al. [59] had screened out new hyperaccumulators (e.g., *T. caerulescens*, *S. alfredii*, and *Bladder campion*), which promoted subsequent scholars to actively apply them to phytoremediation and study their specific mechanisms.

### 3.4. Hotspots and Trends Analysis

#### 3.4.1. Keyword Cluster Analysis

Keywords are a high-level summary and concentrated description of the topic of the article; the keyword clustering analysis is an effective way to present major research domains and topics [60,61]. The clusters and feature information that label the keywords are shown in Table 3 and Figure S2, respectively.

#### Cluster #0: “Biochar”

The first cluster was on biochar, containing 245 keywords, including “biochar”, “biochar properties”, “heavy metal fraction”, “health risk”, and “trace elements”. Biochar is a stable carbon-rich byproduct that is a biomass produced by pyrolysis/carbonization of plants and animals [62]. Through structural and surface modifications of biochar, Zhu et al. [63] found that both surface oxidation and amination improved the adsorption performance of structurally modified biochar for cadmium.

Phytoremediation and the use of biochar are two sound environmental interventions for mitigating soil pollution, and combining them may produce a better restoration effect [22,64]. Contaminated soil in mining areas usually has poor fertility; however, biochar can assist plant growth by enhancing soil fertility and improving phytoremediation efficiency [65]. Amending soil with biochar and arbuscular mycorrhizal inoculants significantly increased the activities of superoxide dismutase (SOD), peroxidase (POD), and catalase (CAT) in the roots, stems, leaves, and ears of maize [66]. Notably, the introduction of new pollutants should be avoided when using biochar to remediate heavy-metal-contaminated soil. For example, biochar prepared from sewage sludge can contain high concentrations of HMs [67].

#### Cluster #1: “*T. caerulescens*”

The second cluster is on *T. caerulescens*, with 230 keywords, including “*T. caerulescens*”, “hyperaccumulation”, “heavy metal”, “environmental pollution”, and “agricultural soils”. This cluster was the earliest research area, in which the mean publication year was 2002. In 1994, Baker et al. [11] reported that *T. caerulescens* had the ability to hyperaccumulate cadmium in shoots. Several studies have been conducted on its mechanisms and applications in phytoremediation [52,68,69].

There are excellent articles on the interpretation of the term hyperaccumulation from the early days to the present [15,20,50]. van der Ent et al. [21] pointed out that “accumulation” should only imply active accumulation inside the plant leaf tissue, via the roots. This is because passive accumulation occurs via airborne deposition on the plant leaves. At the molecular level, several genes are overexpressed under cadmium stress. The overexpression of these genes controls cadmium uptake, translocation from root to shoot, accumulation, sequestration, and detoxification [70,71,72]. Thus, the field of hyperaccumulation has become more comprehensive and systematic.

#### Cluster #2: “Endophytic Bacteria”

“Endophytic bacteria” is the third cluster, with 171 keywords. The main keywords were “endophytic bacteria”, “rhizobacteria”, “plant growth-promoting rhizobacteria (PGRA)”, “arbuscular mycorrhizal fungi” (AMF), and “plant growth promotion”. Endophytic bacteria can alleviate metal toxicity in plants through their own metal resistance systems and promote plant growth under metal stress [73]. Hence, phytoremediation assisted by bacterial endophytes is highly recommended [74,75]. Generally speaking, there are two main ways for endophytic bacteria to improve plant growth in metal-polluted soils: (1) producing plant growth with beneficial substances including solubilization/transformation of mineral nutrients (e.g., phosphate, nitrogen, and potassium), production of phytohormones, siderophores, and specific enzymes; and (2) controlling plant pathogens by inducing a systemic resistance of plants against pathogens [76]. For example, AMF can accumulate high concentrations of HMs and intensify the plant’s ability to tolerate metal stress [77].

#### Cluster #3: “Oxidative Stress”

This cluster focused on oxidative stress, consisting of 147 keywords, including “oxidative stress”, “antioxidant enzymes”, “reactive oxygen species”, “photosynthesis”, and “cadmium stress”. Cadmium can activate reactive oxygen species (ROS) to induce chromosomal aberrations, gene mutations, and DNA damage that affect the cell cycle and division [5,78]. The overproduction of ROS eventually leads to plant physiological disorders [5,79]. In response, plants would produce various molecules and compounds, such as Ca^2+^ [80], glutathione [81], proline [82,83], phytohormones [84,85], and different antioxidant enzymes [86,87,88]. These processes may chelate cadmium, reduce oxidative stress, and reduce damage to plant tissues.

#### Cluster #4: Ethylenediaminetetraacetic Acid (“EDTA”)

“EDTA” is the fifth cluster, consisting of 123 keywords, including “EDTA”, *(S,S)-N,N′-*ethylenediamine disuccinic acid (“EDDS”), “citric acid”, “phytoextraction”, and “chelating agent”. The successful application of plants in the remediation of contaminated soils depends on the phenotype and genotype of the plants. However, interactions between a chelating agent and HMs are also of great importance because of the solubility and availability of metals to plants [15]. Chemical amendments, such as synthetic organic chelates, can enhance phytoextraction by increasing HM bioavailability in soil, thus enhancing plant uptake and the translocation of HMs from the roots to the green parts of tested plants [89]. Among chelates, EDTA, EDDS, and citric acid have been used as viable environmental technologies to enhance the efficiency of phytoremediation [10,17,39,90]. However, the in-situ application of chelating agents can cause groundwater pollution through uncontrolled metal dissolution and leaching. Therefore, the potential risks of groundwater contamination should be thoroughly assessed before using EDTA or other chelators for phytoextraction.

#### Cluster #5: “Bioconcentration Factor”

A total of 66 keywords made up this cluster, and the main keywords were “bioconcentration factor”, “translocation factor”, “subcellular distribution”, “cadmium pollution”, and “chemical form”. This cluster was mainly concerned with the molecular biology mechanisms by which hyperaccumulators absorb, transport, isolate, and tolerate metals. Hyperaccumulators have three basic hallmarks that distinguish them from related non-hyperaccumulating taxa: a stronger enhanced rate of HMs uptake, a faster root-to-shoot translocation and a greater ability to detoxify and sequester HMs in leaves [15]. These processes are driven by the overexpression of gene encoding transmembrane transporters, such as members of the Zn-regulated transporter and iron-regulated transporter-like (ZIP) family, P_1B_-ATPase (HMA) family, yellow stripe-like (YSL) transporter family, and metal tolerance protein (MTP) family [15,91].

A total of 313 HM-associated transporters (HMATs) widely distributed in 17 transporter families, were found to be responsible for HM uptake, transport, and translocation in plants [92]. SaHMA3 showed higher expression in the hyperaccumulating population than in the non-hyperaccumulating population. These findings indicate that SaHMA3h plays an important role in facilitating cadmium sequestration in the vacuole of *S. alfredii* [93]. SaMT2, a type II metallothionein gene, had a higher transcript level in the shoot than in the root, indicating that SaMT2 played an important role in transporting cadmium in *S. alfredii* [94].

#### 3.4.2. Keyword Co-Occurrence Analysis

**Figure 6:**
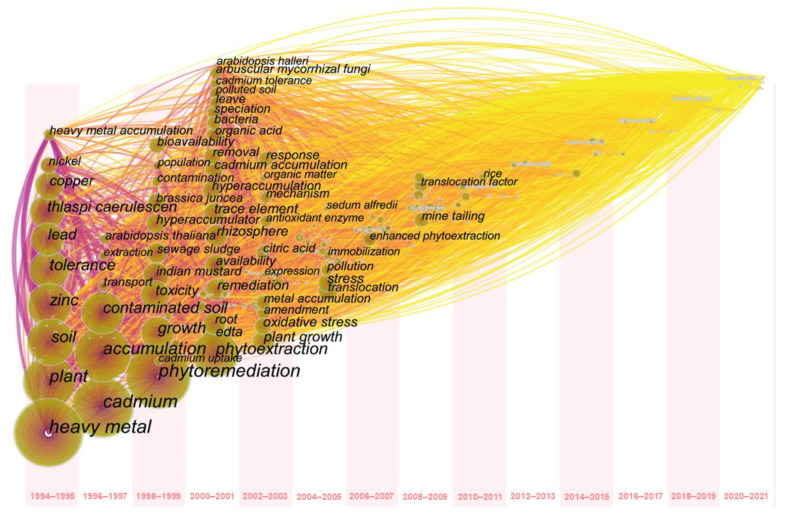
Timezone view of keyword co-occurrence.

A keyword co-occurrence analysis was also conducted using CiteSpace software. The keyword co-occurrence time zone is generally used to demonstrate the development sequence of this field. The node size is proportional to the keyword frequency and year in which the keyword first appeared. The time zone view of the keyword co-occurrence analysis is shown in Figure 6, and the top 20 keywords in terms of frequency and centrality of this field over the past 28 years are shown in Table 4. In this process, keywords with the same meaning were combined, such as “cadmium” with “Cd”, and “heavy metal” with “metal”.

As shown in Table 4, “heavy metal” (3050 times), “cadmium” (2747 times), “phytoremediation” (1886 times), “accumulation” (1707 times), and “zinc” (1492 times) were the most frequent keywords. Some scholars have pointed out that using hyperaccumulators to remove HM from soils was a novel strategy [46,47,95]. Thereafter, scholars have conducted extensive studies to explore the hyperaccumulation mechanism of hyperaccumulators based on cytology, molecular biology, and genetics [14,15,16]. van der Ent et al. [21] outlined the main considerations for establishing the metal or metalloid hyperaccumulation status of plants, (re)defined some of the terminology, and identified potential hazards. These studies have provided standard terminology and methods for hyperaccumulators. Molecular tools have been developed to improve the understanding of the mechanisms by which plants absorb, transport, isolate, and tolerate metals [96,97,98]. Overall, phytoremediation is considered a cost-effective, efficient, novel, and eco-friendly technology, which remains an active field of current research [20,99].

In Figure 6, we demonstrate that the core concepts and basic theories were established around 2011. The work of the past decade (2012–2021) aimed to supplement the existing concepts and deepen them. The development of theoretical knowledge is positively facilitating the application of advanced technologies; however, the pace of theoretical development is relatively slow. Therefore, new ideas and research methods need to be introduced to the phytoremediation of cadmium-contaminated soil to boost innovation and beneficial outcomes.

#### 3.4.3. Keyword Bursts Analysis

Some keywords sharply increased within a specific period, reflecting the dynamics and potential research questions of a field. A keyword burst analysis can be used to analyze development trends and research frontiers. Through CiteSpace, 180 keywords with occurrence bursts from 1994 to 2021 were identified. Table 5 shows the top 30 keywords with the strongest bursts, which were the hottest topics or the most studied research areas. Based on the correlation of these 30 keywords, the field can be divided into the following main aspects.

(1) Screening new hyperaccumulators and applying them in the phytoremediation of cadmium-contaminated soil. Scholars have struggled to find plants with the ability to hyperaccumulate cadmium that can be employed in phytoremediation. These studies were conducted relatively early on. For example, the keyword burst for “*T. caerulescens*” (1997–2011) began in 1997 and ended in 2011; other examples include “*Indian mustard*” (1999–2010) and “*Arabidopsis halleri*” (2000–2011). In addition, scholars later focused on “*Salix*” (2004–2011), “tree” (2005–2011), and “fern” (2007–2012).
(2) Searching for strategies that improve the efficiency of phytoremediation. Researchers improved the efficiency of phytoremediation in multiple ways, such as amending the rhizosphere microbial community with “glomus mosseae” (2004–2011), adding “organic acid” (2004–2010), and adding “EDTA” (2006–2011). The keyword burst for “biochar” and “bacterial community” began in 2019 and continues to date, thereby indicating that biochar and bacterial community are current research hotspots.
(3) Exploring the mechanisms by which these plants hyperaccumulate metal. Exploring the enrichment mechanism of hyperaccumulators can help understand how hyperaccumulators absorb, translocate, accumulate, sequester, and detoxify cadmium. Research in this area began early and continues to this day. The keyword bursts in this aspect included “phytochelatin” (1994–2008), “transport” (1995–2007), “cadmium uptake” (1997–2006), “absorption” (2000–2008), “heavy metal uptake” (2002–2011), and “subcellular distribution” (2018–2021). Among them, “subcellular distribution” began in 2018 and still continues, indicating that much more attention is given to this topic in recent years [100,101].
(4) Assessment of health risks. High concentrations of HMs in the soil are a risk to human health; HMs enter the food chain via agricultural products or contaminated drinking water. Phytoremediation can effectively remove cadmium from cadmium-contaminated soil and eliminate the long-term risk of cadmium entering the food chain. The keyword burst for “health risk” began from 2016 to 2021, indicating that health risk is a new line of research for phytoremediation of cadmium-contaminated soil [45,102].

## 4. Conclusions

This study offered a knowledge mapping of the phytoremediation of cadmium-contaminated soil. Based on the results of the bibliometric analysis, the publication characteristics, research power, and research hotspots in this field were systematically reviewed and presented in this study.

Over the past 28 years, 5494 related scientific articles were published, with nearly half being published in the past five years, indicating that researchers are paying increasing attention to this field. Phytoremediation of cadmium-contaminated soil is a multifaceted and multidisciplinary field, in which environmental science and ecology, environmental science, plant science, agriculture, and engineering were the five major categories. The *International Journal of Phytoremediation* was the most productive journal and *Chemosphere* had the highest co-citation frequency.

Most of the productive authors (e.g., Xiaoe Yang, Shuhe Wei, and Longhua Wu) and institutions (e.g., Chinese Academy of Sciences and Zhejiang University) were from China. However, Baker A.J.M. (Australia) was the most influential author in terms of citation frequency. The most productive countries were mainly located in Asia, North America, and Europe. China had the largest number of publications. However, the USA was the most influential country in terms of centrality. The identified research domains were labeled as “biochar”, “*T. caerulescens*”, “endophytic bacteria”, “oxidative stress”, “EDTA”, and “bioconcentration factor”.

The core concepts and basic theories of this field were basically completed in 2011. Over the ensuing decade, the development and refinement of theory and fundamental knowledge has driven the application of phytoremediation of cadmium-contaminated soil. However, there has been little innovation in the core concepts and fundamental theories in this research field over the past decade. Therefore, this field requires innovation, new perspectives, and methods to further develop phytoremediation of cadmium-contaminated soil.

Combined with the keyword cluster, co-occurrence, and burst analysis, the research trends/frontiers were summarized to provide guidance and reference for scholars.

(1) Improving phytoremediation efficiency by biochar addition and manipulation of the rhizosphere bacterial community. Phytoremediation can remove HMs from soil; however, its efficiency is affected by plant growth speed and biomass. Therefore, it is necessary to integrate various methods, such as combining biochar and amendment with AMF to improve efficiency [22,77,103].
(2) Further investigation of cadmium subcellular distribution. Research in this field will provide a microscopic and multidisciplinary perspective on hyperaccumulation, helping us to better understand how and why hyperaccumulators differ from normal plants. Moreover, it will promote the plant evolution field.
(3) Health risk assessments may become a new line of research. Cadmium-contaminated soil is a risk to human health, as cadmium can enter the food chain via agricultural products. After phytoremediation, the risk of cadmium entering the food chain can be eliminated [104,105]. In recent years, public health has become increasingly concerned with environmental issues. Thus, phytoremediation combined with health risk assessment may become a research hotspot in the future.

Despite the significant and multiple results obtained from the visualization analysis of phytoremediation of cadmium-contaminated soil articles, there were several limitations in this study. The analyzed data were only derived from the WOS Core Collection databases in English, which limited the return results due to linguistic bias and exclusion of other databases. Therefore, more comprehensive and in-depth analyses are needed in the future.

## Figures and Tables

**Table 1:** Top 10 journals, authors, institutions, countries, cited journals, and cited authors from 1994 to 2021.

Rank	Journal Source	Author	Institution	Country	Cited Journal	Cited Author
	(Publications)	(Publications)	(Publications, Country)	(Publications, Centrality)	(Frequency)	(Frequency)
1	*International Journal of Phytoremediation*	Xiaoe Yang	Chinese Acad Sci	China	*Chemosphere*	Baker A.J.M.
	(474)	(72)	(439, China)	(1950, 0.12)	(3801)	(1644)
2	*Environmental Science and Pollution Research*	Shuhe Wei	Zhejiang Univ	USA	*Environmental Pollution*	Salt D.E.
	(429)	(42)	(163, China)	(474, 0.23)	(3644)	(1005)
3	*Chemosphere*	Longhua Wu	Univ Chinese Acad Sci	India	*Plant and Soil*	McGrath S.P.
	(325)	(41)	(108, China)	(415, 0.08)	(3323)	(897)
4	*Ecotoxicology and Environmental Safety*	Shafaqat Ali	Sichuan Agr Univ	Pakistan	*Science of the Total Environment*	Kabata-Pendias A.
	(217)	(41)	(107, China)	(319, 0.07)	(2745)	(745)
5	*Environmental Pollution*	Lijin Lin	Univ Florida	France	*Journal of Hazardous Materials*	Zhao F.J.
	(189)	(40)	(88, American)	(294, 0.10)	(2592)	(600)
6	*Journal of Hazardous Materials*	Yongming Luo	Nanjing Agr Univ	Spain	*Environmental Science & Technology*	Chaney R.L.
	(163)	(36)	(82, China)	(275, 0.14)	(2397)	(555)
7	*Science of the Total Environment*	Ming’an Liao	CSIC	Poland	*International Journal of Phytoremediation*	Clemens S.
	(158)	(34)	(81, Spain)	251, 0.02)	(2315)	(552)
8	*Plant and Soil*	Qixing Zhou	Northwest A&F Univ	Italy	*New Phytologist*	Ali H.
	(158)	(34)	(71, China)	(228, 0.21)	(2081)	(505)
9	*Water Air and Soil Pollution*	Muham Mad Rizwan	Sun Yat Sen Univ	Iran	*Environmental and Experimental Botany*	Lombi E.
	(117)	(34)	(68, China)	(192, 0.03)	(2035)	(490)
10	*Journal of Environmental Management*	Zhenli He	Univ Agr Faisalabad	Germany	*Water Air and Soil Pollution*	Lasat M.M.
	(79)	(32)	(67, Pakistan)	(180, 0.10)	(2005)	(484)

**Table 2:** Top 10 most-cited research articles about phytoremediation of cadmium-contaminated soil.

Publication	Citation Frequency	Journal	Reference
Phytoextraction: the use of plants to remove heavy metals from soils	963	*Environmental Science & Technology*	[47]
Phytoremediation of lead-contaminated soils: role of synthetic chelates in lead phytoextraction	822	*Environmental Science & Technology*	[54]
Hyperaccumulators of metal and metalloid trace elements: facts and fiction	796	*Plant and Soil*	[21]
Heavy metal accumulation and tolerance in British populations of the metallophyte *T. caerulescens* J. & C. Presl (Brassicaceae)	623	*New Phytologist*	[11]
Cadmium tolerance and hyperaccumulation in a new Zn-hyperaccumulating plant species (*S. alfredii* Hance)	566	*Plant and Soil*	[13]
Rhizofiltration: the use of plants to remove heavy metals from aqueous streams	492	*Environmental Science & Technology*	[55]
Accumulation properties of As, Cd, Cu, Pb and Zn by four wetland plant species growing on submerged mine tailings	422	*Environmental and Experimental Botany*	[56]
Phytoremediation potential of *T. caerulescens* and *Bladder campion* for zinc- and cadmium-contaminated Soil	401	*Journal of Environmental Quality*	[57]
Strategies of heavy metal uptake by three plant species growing near a metal smelter	380	*Environmental Pollution*	[58]
Phytoextraction of cadmium and zinc from a contaminated soil	378	*Journal of Environmental Quality*	[59]

**Table 3:** Keyword cluster analysis and feature information.

Cluster ID	Cluster Name	Size	Silhouette	Mean (Year)	Main Keywords
0	Biochar	245	0.572	2010	biochar; biochar properties; heavy metal fraction; health risk; trace elements
1	*T. caerulescens*	230	0.73	2002	*T. caerulescens*; hyperaccumulation; heavy metal; environmental pollution; agricultural soils
2	Endophytic bacteria	171	0.614	2009	endophytic bacteria; rhizobacteria; PGRA; arbuscular mycorrhizal fungi
3	Oxidative stress	147	0.658	2012	oxidative stress; antioxidant enzymes; reactive oxygen species; photosynthesis; cadmium stress
4	EDTA	123	0.622	2009	EDTA; EDDS; citric acid; phytoextraction; chelating agent
5	Bioconcentration factor	66	0.782	2010	bioconcentration factor; translocation factor; subcellular distribution; cadmium pollution; chemical form

**Table 4:** The top 20 keywords related to phytoremediation of cadmium-contaminated soil of 1994 to 2021.

Keyword	Freq	Centrality	Keyword	Freq	Centrality
heavy metal	3050	0.03	growth	775	0.02
cadmium	2747	0.01	tolerance	739	0.03
phytoremediation	1886	0.00	copper	604	0.04
accumulation	1707	0.01	*T. caerulescen*	480	0.02
zinc	1492	0.01	toxicity	470	0.01
plant	1372	0.01	plant growth	385	0.02
contaminated soil	1167	0.04	remediation	319	0.01
soil	1146	0.01	EDTA	258	0.01
lead	1133	0.03	rhizosphere	252	0.01
phytoextraction	1043	0.01	oxidative stress	246	0.01

**Table 5:** Top 30 keywords with the strongest burst.

Keywords	Strength	Begin	End	1994–2021
nickel	14.83	1994	2005	▃▃▃▃▃▃▃▃▃▃▃▃ ▂▂▂▂▂▂▂▂▂▂▂▂▂▂▂▂
phytochelatin	9.34	1994	2008	▃▃▃▃▃▃▃▃▃▃▃▃▃▃▃ ▂▂▂▂▂▂▂▂▂▂▂▂▂
transport	17.72	1995	2007	▂ ▃▃▃▃▃▃▃▃▃▃▃▃▃ ▂▂▂▂▂▂▂▂▂▂▂▂▂▂
zinc	44.15	1996	2008	▂▂ ▃▃▃▃▃▃▃▃▃▃▃▃▃ ▂▂▂▂▂▂▂▂▂▂▂▂▂
*T. caerulescen*	50.36	1997	2011	▂▂▂ ▃▃▃▃▃▃▃▃▃▃▃▃▃▃▃ ▂▂▂▂▂▂▂▂▂▂
population	16.47	1997	2008	▂▂▂ ▃▃▃▃▃▃▃▃▃▃▃▃ ▂▂▂▂▂▂▂▂▂▂▂▂▂
cadmium uptake	14.93	1997	2006	▂▂▂ ▃▃▃▃▃▃▃▃▃▃ ▂▂▂▂▂▂▂▂▂▂▂▂▂▂▂
brassicaceae	11.15	1997	2007	▂▂▂ ▃▃▃▃▃▃▃▃▃▃▃ ▂▂▂▂▂▂▂▂▂▂▂▂▂▂
hyperaccumulator *T. caerulescen*	23.65	1998	2009	▂▂▂▂ ▃▃▃▃▃▃▃▃▃▃▃▃ ▂▂▂▂▂▂▂▂▂▂▂▂
*Indian mustard*	44.11	1999	2010	▂▂▂▂▂ ▃▃▃▃▃▃▃▃▃▃▃▃ ▂▂▂▂▂▂▂▂▂▂▂
absorption	11.96	2000	2008	▂▂▂▂▂▂ ▃▃▃▃▃▃▃▃▃ ▂▂▂▂▂▂▂▂▂▂▂▂▂
*A. halleri*	10.17	2000	2011	▂▂▂▂▂▂ ▃▃▃▃▃▃▃▃▃▃▃▃ ▂▂▂▂▂▂▂▂▂▂
lead phytoextraction	12.1	2001	2010	▂▂▂▂▂▂▂ ▃▃▃▃▃▃▃▃▃▃ ▂▂▂▂▂▂▂▂▂▂▂
metal	8.88	2001	2006	▂▂▂▂▂▂▂ ▃▃▃▃▃▃ ▂▂▂▂▂▂▂▂▂▂▂▂▂▂▂
heavy metal uptake	12.41	2002	2011	▂▂▂▂▂▂▂▂ ▃▃▃▃▃▃▃▃▃▃ ▂▂▂▂▂▂▂▂▂▂
availability	12.73	2003	2008	▂▂▂▂▂▂▂▂▂ ▃▃▃▃▃▃ ▂▂▂▂▂▂▂▂▂▂▂▂▂
*Salix*	10.27	2004	2011	▂▂▂▂▂▂▂▂▂▂ ▃▃▃▃▃▃▃▃ ▂▂▂▂▂▂▂▂▂▂
glomus mosseae	9.29	2004	2011	▂▂▂▂▂▂▂▂▂▂ ▃▃▃▃▃▃▃▃ ▂▂▂▂▂▂▂▂▂▂
organic acid	8.86	2004	2010	▂▂▂▂▂▂▂▂▂▂ ▃▃▃▃▃▃▃ ▂▂▂▂▂▂▂▂▂▂▂
tree	8.72	2005	2011	▂▂▂▂▂▂▂▂▂▂▂ ▃▃▃▃▃▃▃ ▂▂▂▂▂▂▂▂▂▂
EDTA	14.86	2006	2011	▂▂▂▂▂▂▂▂▂▂▂▂ ▃▃▃▃▃▃ ▂▂▂▂▂▂▂▂▂▂
fern	8.56	2007	2012	▂▂▂▂▂▂▂▂▂▂▂▂▂ ▃▃▃▃▃▃ ▂▂▂▂▂▂▂▂▂
extraction	8.29	2009	2011	▂▂▂▂▂▂▂▂▂▂▂▂▂▂▂ ▃▃▃ ▂▂▂▂▂▂▂▂▂▂
health risk	8.94	2016	2021	▂▂▂▂▂▂▂▂▂▂▂▂▂▂▂▂▂▂▂▂▂▂ ▃▃▃▃▃▃
potentially toxic element	8.91	2017	2021	▂▂▂▂▂▂▂▂▂▂▂▂▂▂▂▂▂▂▂▂▂▂▂ ▃▃▃▃▃
subcellular distribution	9.44	2018	2021	▂▂▂▂▂▂▂▂▂▂▂▂▂▂▂▂▂▂▂▂▂▂▂▂ ▃▃▃▃
biochar	12.81	2019	2021	▂▂▂▂▂▂▂▂▂▂▂▂▂▂▂▂▂▂▂▂▂▂▂▂▂ ▃▃▃
remediation	10.64	2019	2021	▂▂▂▂▂▂▂▂▂▂▂▂▂▂▂▂▂▂▂▂▂▂▂▂▂ ▃▃▃
chemical form	9.41	2019	2021	▂▂▂▂▂▂▂▂▂▂▂▂▂▂▂▂▂▂▂▂▂▂▂▂▂ ▃▃▃
bacterial community	9.18	2019	2021	▂▂▂▂▂▂▂▂▂▂▂▂▂▂▂▂▂▂▂▂▂▂▂▂▂ ▃▃▃

Note: The thick red lines indicate the keywords with occurrence bursts in these years.

## Data Availability

The data presented in this study are available on request from the corresponding author. The data are not publicly available due to privacy/ethical restrictions.

## Supplementary Materials

The following supporting information can be downloaded at: https://www.mdpi.com/article/10.3390/ijerph19126987/s1, Figure S1: Journal co-citation network (a) and author co-citation network (b); Figure S2: Main clusters labeled by keyword.
