# Supplementary material for: Knowledge Mapping of the Phytoremediation of Cadmium-Contaminated Soil: A Bibliometric Analysis from 1994 to 2021
Source: Int J Environ Res Public Health. 2022 Jun 7;19(12):6987. doi: 10.3390/ijerph19126987 (PMC9222242; doi:10.3390/ijerph19126987)
Supplement: Supplementary file 1 [file ijerph-19-06987-s001.zip › ijerph-1730198-supplementary.pdf]

Supplementary Materials

Knowledge Mapping of the Phytoremediation of Cadmium-Contaminated Soil: A Bibliometric Analysis from 1994 to 2021

Xiaofeng Zhao [1,2], Mei Lei [1,2,*] and Runyao Gu [3]

[1] Institute of Geographic Sciences and Natural Resources Research, Chinese Academy of Sciences, Beijing 100101, China; zhaoxf.15b@igsnrr.ac.cn
[2] University of Chinese Academy of Sciences, Beijing 100049, China
[3] College of Eco-Environmental Engineering, Guizhou Minzu University, Guiyang 550025, China; gu.ry@gzmu.edu.cn
* Correspondence: leim@igsnrr.ac.cn; Tel.: +86-010-64889115

Contents of this file:

Pages: 2

Number of Figures: 2 (Figures S1 and S2)

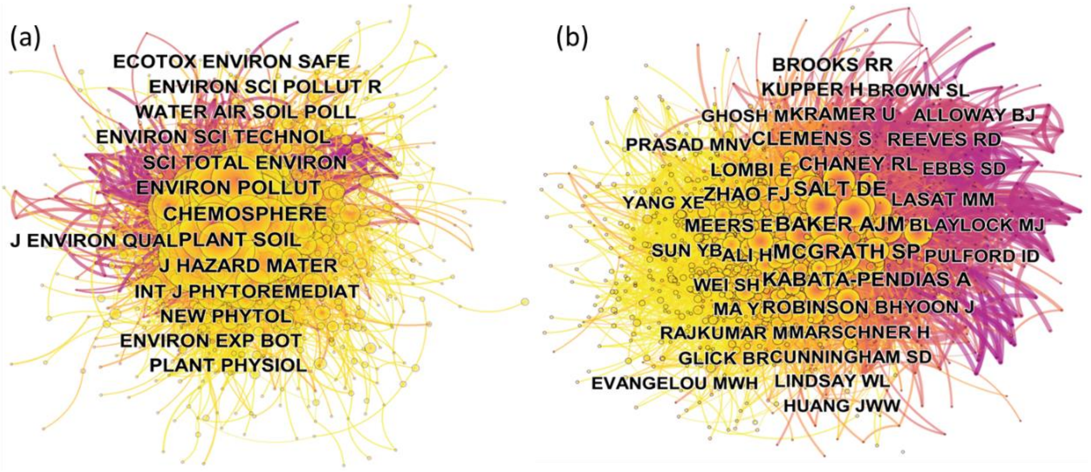

Figure S1. Journal co-citation network (**a**) and author co-citation network (**b**).

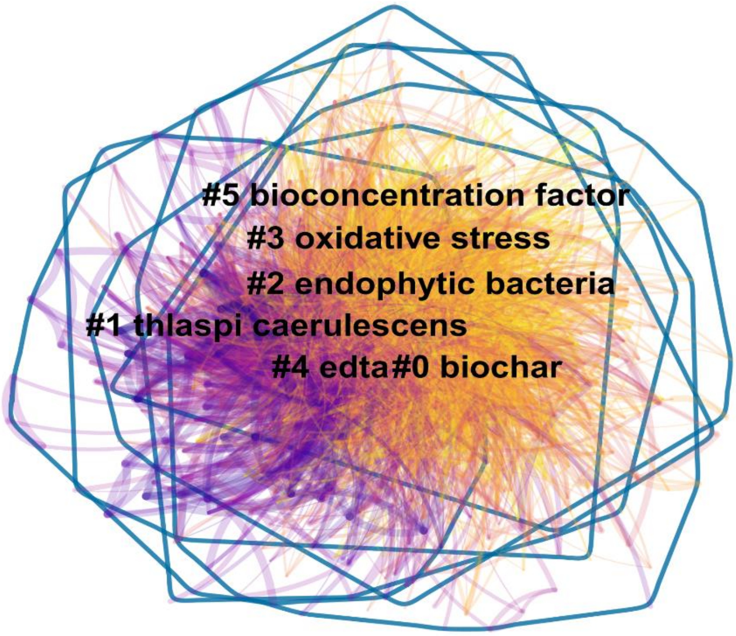

Figure S2. Main clusters labeled by keyword.
